# Atorvastatin inhibits insulin synthesis by inhibiting the Ras/Raf/ERK/CREB pathway in INS-1 cells

**DOI:** 10.1097/MD.0000000000004906

**Published:** 2016-09-30

**Authors:** Hongxi Sun, Yu Li, Bei Sun, Ningning Hou, Juhong Yang, Miaoyan Zheng, Jie Xu, Jingyu Wang, Yi Zhang, Xianwei Zeng, Chunyan Shan, Bai Chang, Liming Chen, Baocheng Chang

**Affiliations:** a Department of Nephrology, 2011 Collaborative Innovation Center of Tianjin for Medical Epigenetics, Key Laboratory of Hormones and Development (Ministry of Health), Tianjin Key Laboratory of Metabolic Diseases, Tianjin Metabolic Diseases Hospital & Tianjin Institute of Endocrinology, Tianjin Medical University, Tianjin, China; b Department of Endocrinology; c Department of Neurosurgery, The Affiliated Hospital of Weifang Medical College, Weifang, Shandong 261031, China.

**Keywords:** CRE, insulin synthesis, Ras complex pathway, statins, type 2 diabetes

## Abstract

**Backround:**

Type 2 diabetes has become a global epidemic disease. Atorvastatin has become a cornerstone in the prevention and treatment of atherosclerosis. However, increasing evidence showed that statins can dose-dependently increase the risk of diabetes mellitus. The mechanism is not clear.

**Objective:**

The Ras complex pathway (Ras/Raf/extracellular signal-regulated kinase [ERK]/cAMP response element-binding protein [CREB]) is the major pathway that regulates the gene transcription. Except for the inhibition of cholesterol synthesis by inhibiting the 3-hydroxy-3-methyl glutaryl coenzyme A (HMG-COA) reductase, statins can also downregulate the phosphorylation of a series of downstream substrates including the key proteins of the Ras complex pathway, therefore may inhibit the insulin syntheses in pancreatic beta cells. In our study, we investigated the inhibitory effect and the underlying mechanism of atorvastatin on insulin synthesis in rat islets.

**Methods:**

Islets were isolated from Wistar rats and cultured in Roswell Park Memorial Institute (RPMI)-1640 medium. The insulin content in the medium was measured by radioimmunoassay before and after the treatment of 50 μM atorvastatin. Effect of atorvastatin on the expression of insulin message Ribonucleic acid (mRNA) in pancreatic islet beta cells was also detected using quantitative real-time polymerase chain reaction. Western blotting was used to explore the possible role of the Ras complex pathway (Ras/Raf/ERK/CREB) in atorvastatin-inhibited insulin synthesis. The effects of atorvastatin on the binding of nuclear transcription factor p-CREB with CRE in INS-1 cells were examined via chromatin immunoprecipitation assay.

**Results:**

Compared with the control group, the insulin level decreased by 27.1% at 24 hours after atorvastatin treatment. Atorvastatin inhibited insulin synthesis by decreasing insulin mRNA expression of pancreatic islet beta cells. The activities of Ras, Raf-1, and p-CREB in the Ras complex pathway were inhibited by 50 μM atorvastatin in INS-1 cells in vitro. Moreover, 50 μM atorvastatin reduced the binding of p-CREB with deoxyribonucleic acid (DNA) in INS-1 cells in vitro.

**Conclusion:**

Atorvastatin inhibits insulin synthesis in beta cells by inhibiting the activation of the Ras complex pathway.

## 1 Introduction

Cardiovascular and cerebrovascular diseases are serious threats to human health. Because of its powerful lipid-regulating effect and good tolerance, atorvastatin has become a cornerstone in the prevention and treatment of atherosclerosis. The 2013 ACC/AHA ASCVD cholesterol treatment guideline^[1]^ proposes the use of moderate- or high-intensity statin therapy in populations with different risks of cardiovascular and cerebrovascular diseases. However, with the wide application of statins, its adverse reactions are also increasing. One latest research evaluated the cost-effectiveness of statins in the elderly aged over 75 years. The results showed that myalgia and mild cognitive impairment were observed in 10% to 29% cases, which offsets the cardiovascular benefits of statins.^[2]^ To prevent and treat cardiovascular diseases, we need an early and comprehensive intervention of the risk factors such as dyslipidemia, hyperglycemia, and so on. Clinical meta-analysis^[3–5]^ showed that statins can dose-dependently increase the risk of diabetes mellitus. The intensive treatment of statins is associated with a higher risk of diabetes, but the mechanism is not clear. A recent clinical study has proved that the increased risk of type 2 diabetes with statin treatment is associated with an impaired insulin sensitivity and insulin secretion.^[6]^ Therefore, it is important to find out how to achieve an effective treatment outcome with statins without significantly affecting blood glucose or other atherosclerotic risk factors.

In addition, type 2 diabetes has become a global epidemic disease; the prevalence of diabetes and prediabetes in China are 11.6% and 50.1%, respectively.^[7]^ By 2035, nearly 4.71 billion people will develop impaired glucose tolerance. Therefore, we need to focus not only on the treatment of diabetes, but also on the control of all the possible risk factors.

Therefore, further studies on the mechanism of statins in the development of new-onset diabetes mellitus have important clinical significance. Our previous studies have found that statins inhibit glucose-stimulated insulin secretion (GSIS) by inhibiting adenosine triphosphate (ATP) formation and ATP-sensitive potassium channel activity in pancreatic islet cells.^[8,9]^ Zhou et al^[10]^ found that the inhibition effect of GSIS was related to the increased expression of Kir6.2 and decreased expression of Cav1.2 and GLUT2 in MIN6 cells.

Since statins are known to inhibit the secretion of insulin, they could probably affect insulin synthesis; however, the underlying mechanism needs to be elucidated. In the insulin signaling pathways, the Ras complex pathway (Ras/Raf/extracellular signal-regulated kinase [ERK]/cAMP response element-binding protein [CREB]) is the main pathway that mediates the transcription of insulin-regulated genes and promotes the growth and proliferation of cells. In addition to inhibiting cholesterol synthesis, statins also reduce the synthesis of other intermediate products of the mevalonate pathway (MVA), the effect of Ras activity, and the intracellular signal transduction. Therefore, to further explore the mechanism of statins, the effect of atorvastatin on insulin synthesis and the underlying mechanism were investigated in the present study.

## 2 Materials and methods

### 2.1 Cell culture and intervention

Rat INS-1 β cells were provided by the Institute of Basic Science, Medical University of Tianjin. The cells were cultured under standard cell culture conditions in Roswell Park Memorial Institute (RPMI)-1640 medium (Hyclone, Logan, UT), containing 11.1 mM glucose, 1 mM sodium pyruvate, 50 μM β-mercaptoethanol, 2 mM l-glutamine, 10 mM hydroxyethyl piperazine ethanesulfonic acid (HEPES), 10% fetal bovine serum (FBS), 100 U/mL penicillin, and 100 μg/mL streptomycin. Subcultures were established twice every week by using trypsin/ethylene diaminetetra-acetic acid (EDTA) treatment. Differentiated INS-1 cells were used for the experiment. Cells were then cultured in RPMI-1640 medium containing 5.6 or 25 mM glucose (5.6 and 25 G, respectively) with/without the intervention of atorvastatin for 2, 6, or 12 hours, and harvested for the assays described below. In some groups, subcultures were pretreated with manumycin A (Sigma, Ronkonkoma, NY) to inhibit GTPase activation.

### 2.2 Preparation of murine pancreatic islets

All animal husbandry and animal experiments were performed according to the Regulations for Animal Experiments and were approved by the Animal Ethics Committee of Tianjin Medical University. Pancreatic islets from Wistar rat were isolated by injecting enzymatic collagenase into the bile duct described elsewhere.^[11]^ Thereafter, the islets were incubated for 2 hours at 37 °C in a 5% CO_2_ atmosphere in RPMI-1640 medium supplemented with 10% FBS, 100 U/mL penicillin, and 100 μg/mL streptomycin. Islets were cultured in RPMI-1640 medium with/without atorvastatin intervention and harvested for the following experiments.

### 2.3 Glucose-stimulated insulin secretion assay

The freshly isolated islets were preincubated with RPMI containing different concentrations (2.8, 5.6, 11.1, 16.7, and 25 mM) of glucose in Krebs-Ringer bicarbonate (KRB) buffer at 37 °C for 30 minutes before measuring secretion. The KRB buffer contained 119 mM NaCl, 4.6 mM KCl, 5 mM NaHCO_3_, 2 mM CaCl_2_, 1 mM MgSO_4_, 0.15 mM Na_2_HPO_4_, 0.4 mM KH_2_PO_4_, 20 mM HEPES, 0.05% bovine serum albumin (BSA), and pH 7.4. Subcultures were subjected to 50 μM atorvastatin for 24 hours. Freshly isolated rat islets were incubated with different concentrations of atorvastatin (0, 10, 30, 50, 100, 200, and 300 μM) as well as high glucose (25 mM glucose concentration) for 24 hours. Supernatants were then obtained. Insulin levels were measured using insulin radioimmunoassay kit (Shibayagi Co., Ltd., Gunma, Japan) and were normalized to the protein concentration, which was determined using a bicinchoninic acid assay.

### 2.4 Insulin assessment

The islets were lysed by using acid ethanol extraction.^[11]^ The freshly isolated rat islets or 24-hour cultured islets of 200 to 250 μm diameter were selected and grouped. Before glucose stimulation, 10 pancreatic isolated islets were preincubated for 24 hours in 11.1 mM glucose. In the experiments of atorvastatin intervention, islets were cultured for 24 hours in RPMI-1640 medium containing 10% fetal calf serum with or without atorvastatin (50 μM) at 37 °C in humidified air containing 5% CO_2_. To determine the insulin concentration, islets were homogenized in 400 μL acid ethanol (37% HCl in 75% ethanol, 15:1000 v/v) and extracted at 4 °C overnight. The acidic extracts were dried by vacuum, reconstituted, and subjected to insulin measurement. Insulin was measured by radioimmunoassay (RIA) (Shibayagi Co., Ltd.).

### 2.5 Drug washout experiments

The islets that were incubated in atorvastatin for 24 hours were cultured for another 24 hours with 11.1 mM glucose in the absence of atorvastatin for 24 hours. The insulin concentration of the supernatant was measured.

### 2.6 Quantitative real-time PCR

Total ribonucleic acid (RNA) was extracted from pancreatic islet beta treated with drug or vehicle for 24 hours by using the TRIzol reagent (Invitrogen, Carlsbad, CA). Isolated RNA (1 μg) was used for the reverse transcriptase reaction with oligo (dT) 18 primers by employing the First-Strand complementary deoxyribonucleic acid (cDNA) synthesis kit (Invitrogen). The insulin message Ribonucleic acid (mRNA) levels were determined by polymerase chain reaction (PCR) (50 cycles, annealing temperature 62 °C) using specific primers (forward: 5′-TCCTGCCCCTGCTGGCCCTGC-3′, reverse: 5′-AGTTGCAGTAGTTCTCCG-3′). Real-time PCRs were performed by monitoring the increase in fluorescence of Synergy Brands (SYBR) Green dye using a real-time detection system. Glyceraldehyde-3-phosphate dehydrogenase expression was used as an internal control. Relative mRNA levels were determined using the ΔΔCt method.

### 2.7 Western blotting

Cells in each group were collected and used to extract total cytoplasmic and nuclear protein. Protein extracts were prepared using the Subcellular Proteome Extraction Kit (Gibco, Life Sciences Solutions Group, USA), and protein concentrations were measured using a protein quantification kit (Gibco). The activity of Ras was assessed using the Ras activation assay kit (Gibco). Equal amounts of protein samples were resolved by sodium dodecyl sulfate (SDS)-polyacrylamide gel electrophoresis and transferred to polyvinylidene fluoride membranes (Merck Millipore, Life Sciences Business Company, Germany). Membranes were then immunoblotted with specific antibodies as follows: rabbit monoclone anti-Ras (1:1000; Gibco), rabbit monoclone anti-Raf (1:1000; Gibco), rabbit monoclone anti-Phospho-Raf (Ser338) (1:1000; Gibco), rabbit monoclone anti-CREB (1:1000; Gibco), and rabbit monoclone anti-Phospho-CREB (Ser133) (1:1000; Gibco). The membranes were then incubated with horseradish peroxidase-immunoglobulin G (IgG) conjugated secondary antibodies and visualized using an enhanced chemiluminescence system (XAR-5 Amersham Life Science, Arlington Height, IL).

### 2.8 Chromatin immunoprecipitation

INS-1 cells which were collected at different times were treated with 1% formaldehyde solution at 37 °C for 10 minutes. Cell precipitates were washed using 1 mL FA Lysis buffer and treated with ultrasound (750 W, 4.5 seconds impact, time interval 9 seconds) for 14 times; the supernatant was then collected by centrifugation. The deoxyribonucleic acid (DNA)–protein antibody complex was formed using target protein-specific antibodies (p-CREB [Ser133]; Sigma) and was precipitated to specifically enrich the DNA fragment. After decoupling the protein and DNA, the DNA fragment was purified and treated with PCR (30 cycles, annealing temperature 58 °C) using specific primers (forward: 5′-ACTGCTTCATCAGGCCATCTG-3′, reverse: 5′-AGGGCTCTAGGAGGGGTAGGT-3′) method. Thus, the target protein–DNA interaction was investigated.

### 2.9 Statistical analysis

All statistical analyses were conducted using SPSS 16.0 statistical software (SPSS Inc., Chicago, IL). All data are expressed as mean ± standard deviation. Unpaired or paired Student *t* test or single factor analysis of variance was used. A *P* value <0.05 was considered statistically significant.

## 3 Result

### 3.1 Inhibitory effect of atorvastatin on islet GSIS and the dose–effect relationship

**Figure 1:**
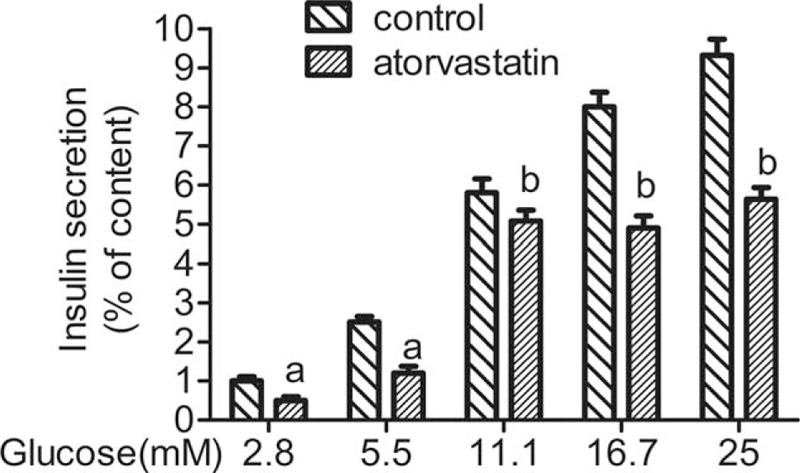
Effect of different concentrations glucose on the glucose-stimulated insulin secretion in rat islets during 24 hours. The freshly isolated islets were pretreated in Krebs-Ringer bicarbonate buffer with different concentrations (2.8, 5.6, 11.1, 16.7, and 25 mM) of glucose. Subcultures were subjected to 50 μM atorvastatin for 24 hours. Supernatants were then collected. Insulin levels were measured. ^a^*P* < 0.05 versus the control group; ^b^*P* < 0.01 versus control group.

**Figure 2:**
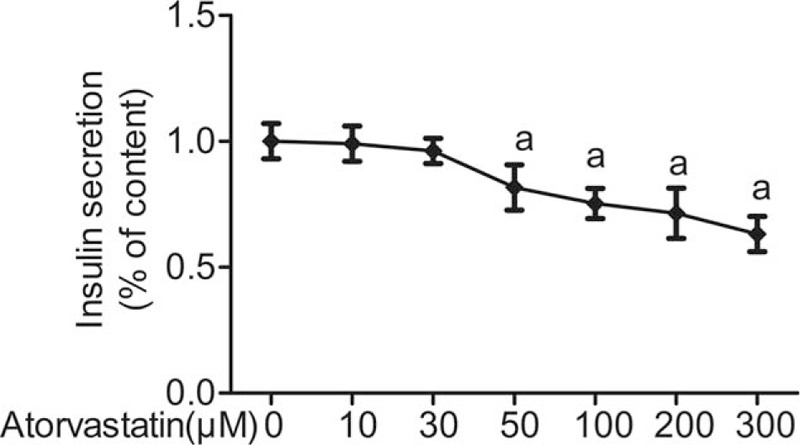
Effect of different concentrations atorvastatin on the glucose-stimulated insulin secretion in rat islets during 24 hours. Freshly isolated rat islets were incubated with different concentrations (0, 10, 30, 50, 100, 200, and 300 μM) atorvastatin (25 mM glucose concentration) for 24 hours. Observe the stimulation of glucose insulin secretory response changes. ^a^*P* < 0.05 versus 0 μM atorvastatin group.

As shown in Fig. 1, freshly isolated rat islets were incubated with 50 μM atorvastatin for 24 hours. Insulin levels were quantified using RIA. After the overnight incubation of atorvastatin for 24 hours, insulin secretion was inhibited by different concentrations of glucose (2.8, 5.6, 11.1, 16.7, and 25 mM) (Fig. 1). Freshly isolated rat islets were incubated with different concentrations of atorvastatin (0, 10, 30, 50, 100, 200, and 300 μM) and high glucose (25 mM glucose concentration) for 24 hours. With the increase of atorvastatin concentration, the inhibitory effect of atorvastatin on islet GSIS was increased in dose-dependent manner. A total of 50 μM atorvastatin treatments caused 40% decrease in GSIS (Fig. 2).

### 3.2 Atorvastatin-inhibited insulin synthesis in pancreatic islet cells

**Figure 3:**
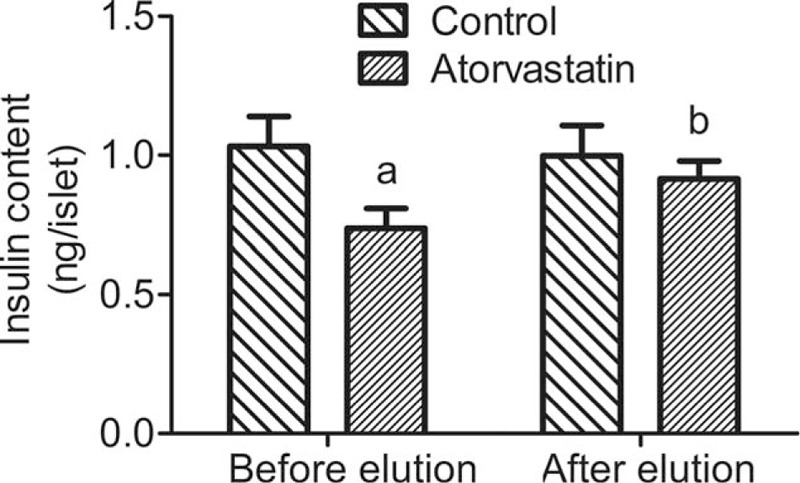
Effect of atorvastatin on the insulin content of islets in rats. Fresh isolated rat islets were cultured for 24 hours in the presence of 50 μM atorvastatin. After 24 hours, fresh isolated rat islets were then analyzed by radioimmunoassay (RIA) or further cultured in medium containing Krebs-Ringer bicarbonate buffer for drug elution. The insulin content of the medium was measured before and after the elution by RIA. ^a^*P* < 0.01 versus the control group; ^b^*P* < 0.01 versus the before elution atorvastatin group.

Freshly isolated rat islets were cultured for 24 hours in the presence of 50 μM atorvastatin, and the changes in insulin concentration in pancreatic islets were measured. Compared with the control group, the insulin content was decreased by 27.1% at 24 hours after atorvastatin treatment (Fig. 3). Moreover, the recovery of islet function was investigated through the washout experiment. The inhibitory effect of statins on insulin concentration was examined. In washout experiment, the insulin level was restored to 90.9% of the control group (Fig. 3).

### 3.3 Atorvastatin inhibited the expression of insulin mRNA in pancreatic islet beta cells

**Figure 4:**
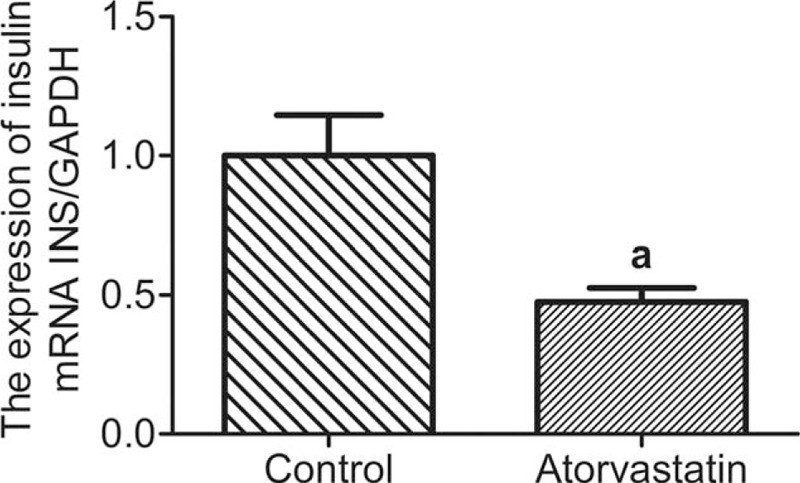
Effect of atorvastatin on the mRNA expression of insulin in pancreatic islet beta cells. Fresh isolated rat pancreatic islet beta cells were cultured in medium containing 50 μM atorvastatin for 24 hours. Then, cell total RNA was harvested and analyzed by quantitative real-time PCR. ^a^*P* < 0.01 versus the control group. mRNA = message Ribonucleic acid, RNA = ribonucleic acid.

Effect of atorvastatin on the mRNA expression of insulin in pancreatic islet beta cells was also studied. Freshly isolated rat pancreatic islet beta cells were cultured in medium containing 50 μM atorvastatin for 24 hours. As shown in Fig. 4, the mRNA expression of insulin was significantly inhibited in pancreatic islet in the treatment group compared to the control group.

### 3.4 Atorvastatin modulated insulin synthesis through the Ras complex pathway (Ras/Raf/ERK/CREB) in INS-1 cells

Ras complex pathway is the main pathway that regulates the gene transcription. The activation of Ras is the first step of this pathway. Activated Ras can activate a series of downstream substrates of the Ras complex pathway (Ras/Raf/ERK/CREB) in INS-1 cells in vitro. CREB functions as a downstream target. Thus, the possible role of the Ras complex pathway (Ras/Raf/ERK/CREB) in the insulin synthesis modulated by atorvastatin was discussed.

### 3.5 Time- and dose-dependent effect of atorvastatin

**Figure 5:**
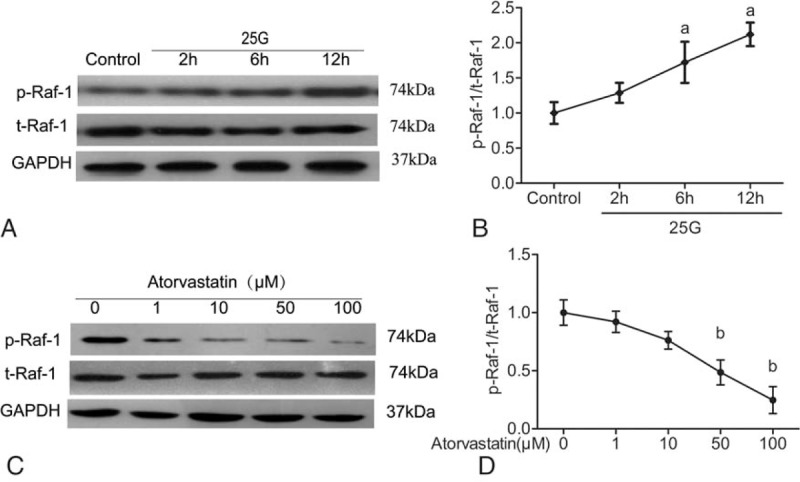
Effect of high glucose on the Ras complex pathway (Ras/Raf/extracellular signal-regulated kinase/CREB) in INS-1 cells in vitro. (a) INS-1 cells were stimulated with or without 25 mM glucose at different times (1, 2, 6, and 12 hours). Then, phosphorylated and total Raf-1 proteins were harvested and detected by Western blotting (A). (b) The bar graphs show the quantification of the indicated proteins (B). (c) INS-1 cells were pretreated with 25 mM glucose for 6 hours. Subcultures were subjected to different concentrations (1, 10, 50, and 100 μM) of atorvastatin with 25 mM glucose for 24 hours. Then, phosphorylated and total Raf-1 proteins were harvested and detected by Western blotting (C). (d) The bar graphs show the quantification of the indicated proteins (D). ^a^*P* < 0.01 versus the control group. ^b^*P* < 0.01 versus the 0 μM atorvastatin group. CREB = cAMP response element-binding protein.

Raf-1 plays an important role in the Ras signaling pathway. Whether Raf-1 can be activated or not is essential for cell function. In the preliminary experiment, the cytosolic Raf-1 level was relatively high and was easily detected. Hence, Raf-1 was chosen as an index to determine the time and the concentration of atorvastatin. INS-1 cells were pretreated with high glucose, collected at different time points (first, second, sixth, and 12th hour), and examined by Western blotting. As shown in Fig. 5A and B, the expression of p-Raf-1 in the high-glucose (25 mM) group was significantly higher than that of the control group, and the increase was time-dependent. After 2 hours, the expression began to increase, with the obvious increase at 6 hours. The expressions at 2, 6, and 12 hours were 1.6, 2.4, and 2.6 times of the control group, while the total amount of Raf-1 was not significantly different.

INS-1 cells were pretreated with 25 mM glucose for 6 hours. Subcultures were subjected to different concentrations of atorvastatin (1, 10, 50, and 100 μM) for 24 hours. Results from Western blotting showed that the Raf-1 activity of the treated cells was inhibited in a concentration-dependent manner at 24 hours. The p-Raf-1 expression did not change in 1 and 10 μmol/L group (Fig. 5C and D); however, it was significantly decreased in 50 μmol/L group, with the maximal inhibition in 100 μmol/L atorvastatin group (Fig. 5C and D). Therefore, atorvastatin inhibited the activation of the key factors in the Ras signaling pathway in a dose-dependent manner induced by high glucose. In addition, atorvastatin had no effect on the expression of total Raf-1 protein in INS-1 cells in vitro.

### 3.6 Atorvastatin decreased the activity of Ras of the Ras complex pathway in INS-1 cells

**Figure 6:**
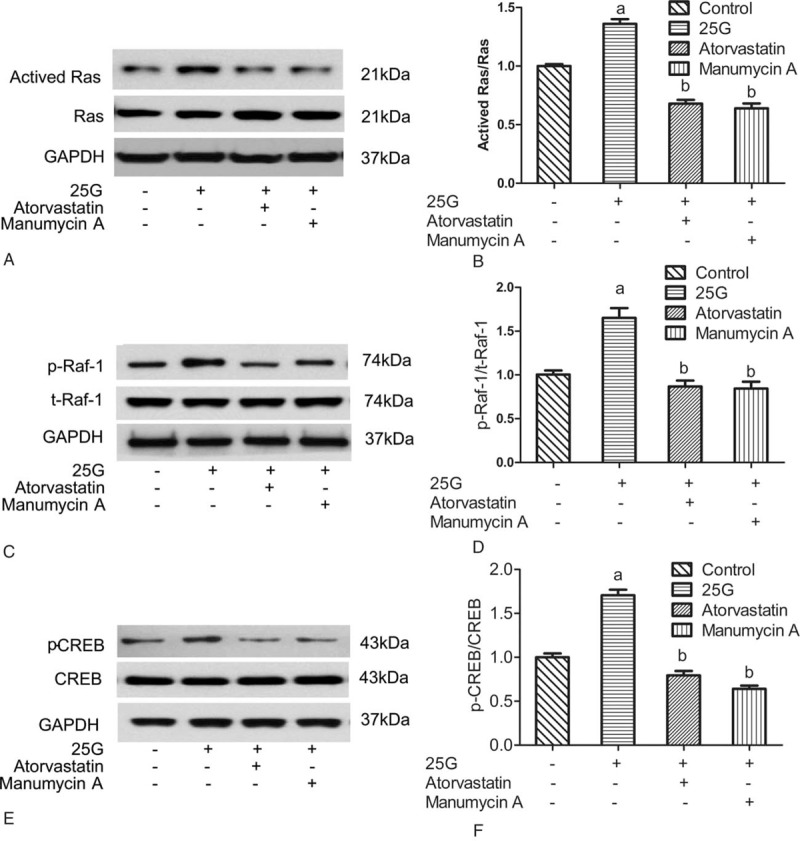
Effect of atorvastatin on the expression and phosphorylation of proteins of the Ras complex pathway (Ras/Raf/extracellular signal-regulated kinase/CREB) in INS-1 cells in vitro. (a) INS-1 cells were pretreated with 50 μM atorvastatin or manumycin A, respectively, and subjected to high glucose (25 mM) for 6 hours. Then Ras activity was harvested and detected by Western blotting (A). (b) The bar graphs show the quantification of the indicated proteins (B). (c) The effect of atorvastatin on downstream critical factors of the Ras complex pathway was evaluated. INS-1 cells were pretreated with 50 μM atorvastatin or manumycin A, respectively, and subjected to high glucose (25 mM) for 6 hours. Then, phosphorylated and total Raf-1 proteins were harvested and detected by Western blotting (C). (d) The bar graphs show the quantification of the indicated proteins (D). (e) INS-1 cells were pretreated with 50 μM atorvastatin or manumycin A, respectively, and subjected to high glucose (25 mM) for 6 hours. Then, phosphorylated and total CREB proteins were harvested and detected by Western blotting (E). (f) The bar graphs show the quantification of the indicated proteins (F). ^a^*P* < 0.01 versus the control group. ^b^*P* < 0.01 versus the 25-G group. CREB = cAMP response element-binding protein.

The activation of Ras is the first step in the Ras complex pathway. Manumycin A, which is a GTPase inhibitor, was used as a positive control in this study. INS-1 cells were pretreated with 50 μM atorvastatin or manumycin A, respectively, and then subjected to high glucose (25 mM) for 6 hours. Results of Western blotting showed that Ras activity in high-glucose group was significantly higher than that of the control group, and the Ras activity in atorvastatin group and manumycin A group was significantly decreased compared with the control group. A total of 50 μM atorvastatin and manumycin A inhibited Ras activity induced by high glucose (Fig. 6A and B); the inhibition rate was 61.8% and 68.7%, respectively. Therefore, the Ras activity was inhibited by 50 μM atorvastatin in INS-1 cells in vitro.

### 3.7 Atorvastatin decreased the activity of p-Raf-1 in the Ras complex pathway in INS-1 cells

INS-1 cells were pretreated with 50 μM atorvastatin or manumycin A, respectively, and subjected to high glucose (25 mM) for 6 hours. Western blotting test showed that the expression levels of p-Raf-1 in atorvastatin group and manumycin A group were significantly decreased compared to those in high-glucose (25 mM) control group. It showed that atorvastatin and manumycin A inhibited the high-glucose-induced increase in the expression of p-Raf-1 (Fig. 6C and D). The inhibition rate was 60.9% and 74.6%, respectively. These results suggested that 50 μM atorvastatin had inhibitory effect on the activity of Raf-1 in the Ras complex pathway.

### 3.8 Atorvastatin decreased the activity of p-CREB of the Ras complex pathway in INS-1 cells in vitro

CREB phosphorylation and activation are essential for its binding with CRE. If the phosphorylation level of CREB is decreased, the combination of p-CREB and DNA will be affected. INS-1 cells were pretreated with 50 μM atorvastatin or manumycin A, respectively, and subjected to high glucose (25 mM) for 6 hours. The expression levels of p-CREB in atorvastatin group and manumycin A group were significantly decreased (*P* < 0.05) (Fig. 6E and F), and the inhibition rates were 55.4% and 64.7%, respectively. Results indicated that 50 μM atorvastatin significantly inhibited the activity of p-CREB in the Ras complex pathway.

### 3.9 Atorvastatin decreased the insulin mRNA expression by reducing the binding of p-CREB with DNA in INS-1 cells in vitro

**Figure 7:**
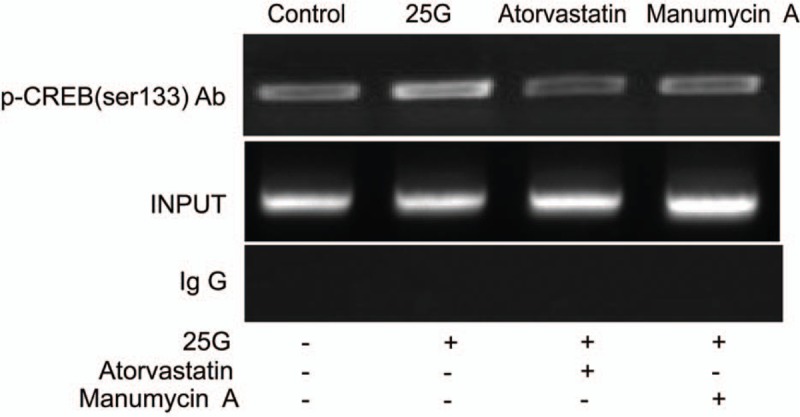
Effect of atorvastatin on the activities of the binding of nuclear transcription factors CREB proteins and *CRE* gene. INS-1 cells were pretreated with 50 μM atorvastatin or manumycin A, respectively, and subjected to high glucose (25 mM) for 6 hours. The effect of atorvastatin on the binding of nuclear transcription factor p-CREB and cAMP response element in INS-1cells was examined via chromatin immunoprecipitation assay–polymerase chain reaction. CREB = cAMP response element-binding protein.

By regulating p-CREB, atorvastatin reduced the binding of p-CREB with DNA in INS-1 cells. As the only specific response element for insulin gene, CRE activation is essential for its binding with p-CREB. INS-1 cells were pretreated with 50 μM atorvastatin or manumycin A, respectively, and subjected to high glucose (25 mM) for 6 hours. The effect of atorvastatin on the binding of nuclear transcription factor p-CREB with CRE in INS-1cells was examined via chromatin immunoprecipitation assay (CHIP)-PCR. The template was divided into 3 parts. The input group template was the genomic DNA fragment, which contained all the *DNA* genes. The negative control template was precipitated by mice-related IgG, and the insulin promoter region was not included. p-CREBAb group template contained insulin promoter region and was bound with CREB antibody after the crosslinking between CREB and genes. CRE primers were amplified in the Ab p-CREB groups and INPUT groups, while the negative control was not amplified. Among the p-CREBAb groups, INS-1 cells were pretreated with 50 μM atorvastatin or manumycin A, respectively, and subjected to high glucose (25 mM) for 6 hours. CHIP detection displayed that the binding of p-CREB with DNA was significantly increased after the stimulation of high glucose (25 mM) in INS-1 cells. Compared with that in high-glucose (25 mM) control group, the binding of p-CREB with DNA in 50 μM atorvastatin treatment group and manumycin A treatment group were significantly decreased in INS-1 cells (Fig. 7). These data indicated that 50 μM atorvastatin reduced the binding of p-CREB with DNA after high glucose stimulation (25 mM) in INS-1 cells in vitro.

## 4 Discussion

Increasing evidence suggests that statins may increase the risk of new-onset diabetes, but the mechanism is not clear. Insulin resistance and secretion dysfunction of pancreatic beta cells are the 2 most important pathophysiological mechanisms in the pathogenesis of type 2 diabetes.

Statins inhibit insulin secretion function of islet beta cells. GSIS relates to the inhibition of ATP-sensitive potassium channel (KATP) and L type calcium channel activity.^[12,13]^ There are various factors that influence the synthesis and secretion of insulin simultaneously. Our study revealed that atorvastatin impaired basal and GSIS and intracellular insulin content. In our study, GSIS of the islets were decreased after 50 μM atorvastatin treatment. In addition, the inhibitory effect of atorvastatin on islet GSIS was dose-dependent. In this study, the effect of atorvastatin on insulin synthesis of pancreatic islet cells was also observed. The cells were cultured overnight with 50 μM atorvastatin for 24 hours, and the content of insulin decreased by 27.1% compared with the control group. In order to further verify that this inhibition is related to statins, the drug washout experiment was carried out and the results showed that the inhibition was significantly restored after 24 hours elution (93.8% of the control group). The results of fluorescence quantitative PCR further confirmed that the expression of insulin mRNA in pancreatic islet beta cells of rats was significantly inhibited by 50 μM atorvastatin after 24 hours. This further supports that statins exhibit varying degrees of inhibition on insulin synthesis.

The mechanism underlying the inhibitory effect of atorvastatin is not known. To the best of our knowledge, this is the first report demonstrating the mechanism of atorvastatin in inhibiting insulin synthesis. The biosynthesis of insulin is a complex process, which is influenced by many factors and multiple links. It was reported that in the physiological concentration range, glucose may stimulate the activity of the insulin promoter and promote the transcription of insulin.^[14,15]^ Exogenous insulin had no effect on the activity of insulin promoter. Further studies found that the increase in the secretion of insulin (such as the effect of potassium chloride) or decrease (such as calcium antagonists) affects neither the promoter activity, nor its sensitivity to glucose stimulation. Shinozuka et al^[16]^ found that a strong inhibitor of protein kinase (especially protein kinase C, PKC) staurosporine may affect insulin gene expression by acting on the cAMP response element (CRE). Thus, it is speculated that glucose may affect the activity of insulin promoter by affecting the signal transduction in pancreatic beta cells, and this effect is not directly related to the changes of intracellular calcium and insulin secretion.

**Figure 8:**
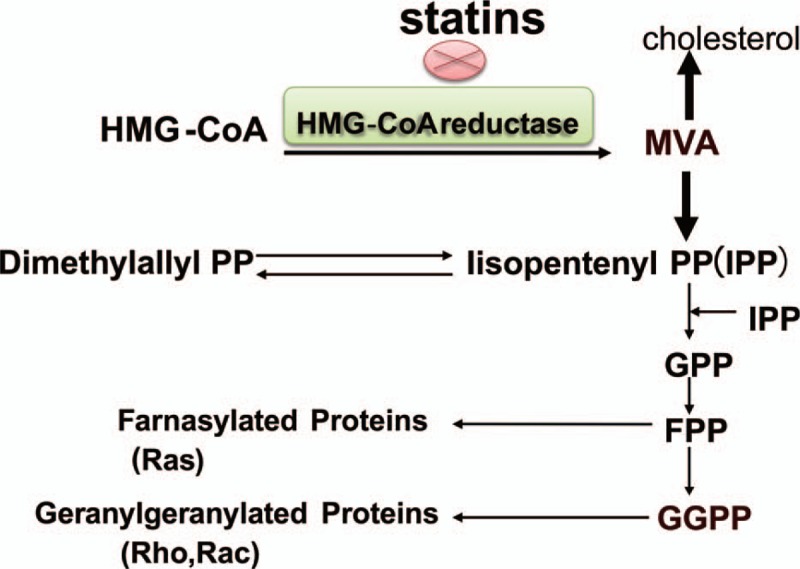
Statins inhibit the mevalonate metabolic pathway. Statins can reduce other intermediate metabolites of the mevalonate pathway metabolic pathway except inhibition of cholesterol synthesis by inhibiting the HMG-CoA reductase, such as farnesyl pyrophosphate ester and dragon bull Quiron geraniol pyrophosphate synthesis. HMG-COA = 3-hydroxy-3-methyl glutaryl coenzyme A.

The Ras complex pathway is the main pathway that regulates gene transcription and promotes cell growth and proliferation. It also participates in metabolic regulation. Ras proteins are anchored to the inner cell membrane. In the insulin signaling pathway, the activated insulin receptor activates the receptor substrate protein, leading to the transduction of the signal to the adapter protein—growth factor receptor binding protein 2 (Grb2). The interaction of Grb2 and signal protein guanosine diphosphate (GDP)–guanosine triphosphate (GTP) exchange factor activates Ras. Active Raf-1 activates the Ras kinase (proto oncogene Raf encoding's serine/threonine kinase), which then activates mitogen-activated protein kinase (MAPK) kinase, also known as MEK, by serine phosphorylation. MEK activates MAPK via phosphorylation. ERK activates 90 kD ribosomal S6 kinase (p90rsk). Then p90rsk will activate CREB and allow it bound to CRE to improve the transcription activity of downstream genes. By inhibiting the 3-hydroxy-3-methyl glutaryl coenzyme A (HMG-COA) reductase, statins can reduce cholesterol synthesis as well as other intermediate metabolites of the MVA metabolic pathway, such as farnesyl pyrophosphate ester (FPP) and dragon bull Quiron geraniol pyrophosphate (geranylgeranyl pyrophosphate [GGPP]). Without GGPP and FPP modification, G protein cannot locate to the cell membrane, which inhibits the signal transduction in cells (Fig. 8). It is suggested that statins may affect insulin synthesis by inhibiting the Ras pathway in the insulin signaling pathway.

In this study, different concentrations of glucose were used in INS-1 cells. The effect of high concentration (25 mM) of glucose on Ras signal transduction pathway was detected by Western blotting in INS-1 cells. It was found that the key factors of the signal transduction pathway were induced under high-glucose conditions. The Raf-1 activity of INS-1 cells was activated by high glucose, and these effects were time-dependent. The key enzyme activity was significantly enhanced in INS-1 cells cultured under high-glucose conditions for 6 hours.

The antiatherosclerosis effect of statins is related to its dose.^[17]^ In the previous study, we had found that the effect of atorvastatin on the inhibition of insulin secretion occurred at concentrations greater than 50 μM after 30 minutes. In the present study, we compared the effects of different concentrations of atorvastatin (1, 10, 50, and 100 μM) on the Raf-1 activity in INS-1 cells. The results showed that the survival status of the cells was not significantly affected by atorvastatin; the phosphorylation activity of the key factors in the signal transduction pathway was inhibited at 10 μM, and the signal transduction pathway was inhibited at 50 μM. With the increasing concentration of atorvastatin, the activity of key factors was inhibited in a dose-dependent manner. Therefore, in order to further study the effects of atorvastatin on Ras pathway (Ras/Raf/ERK/CREB), the intervention concentration and time of atorvastatin were 50 μM and 6 hours, respectively.

Ras pathway of insulin starts with the activation of Ras. When the Ras proteins are activated, they participate in signal transduction and further activate the downstream signal proteins. We confirmed that high concentrations of glucose stimulated the expression of key factors in Ras signal transduction pathway in INS-1 cells. In INS-1 cells treated with 50 μM atorvastatin, the activation of Ras was significantly inhibited. The GTPase inhibitors were used to confirm the inhibition. On the basis of these results, it is concluded that atorvastatin may inhibit the downstream signaling molecule of Ras complex pathway; therefore, the changes of Raf-1 and nuclear transcription factor CREB activity in INS-1 cells were further detected. Studies demonstrated that the inhibitions occurred at the protein level. Therefore, the effect of atorvastatin on the Ras complex pathway transduction pathway is deduced.

**Figure 9:**
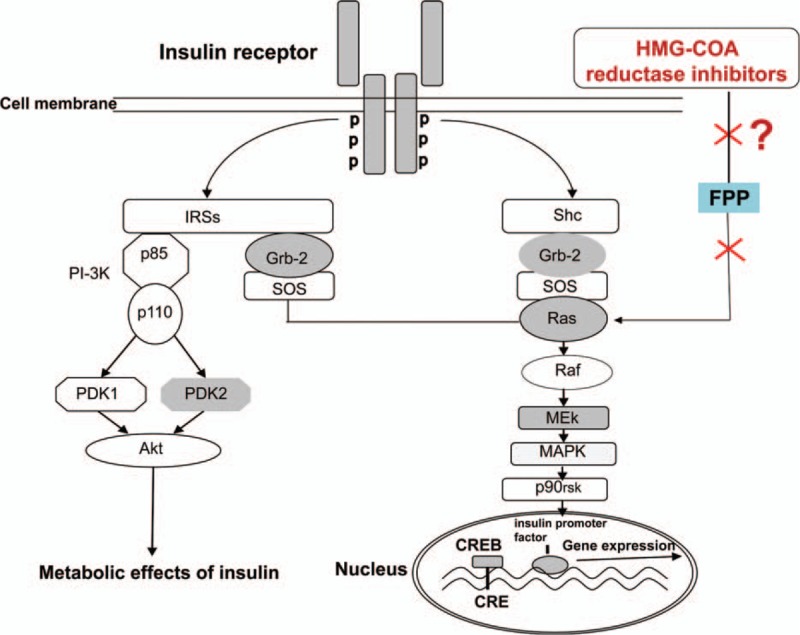
Atorvastatin inhibits insulin synthesis by inhibiting the Ras complex (Ras/Raf/extracellular signal-regulated kinase/CREB) in INS-1 cells. HMG-CoA reductase inhibitor inhibited Ras proteins by inhibiting the generation of farnesyl pyrophosphate ester, and reduced the phosphorylation of a series of downstream substrates, such as Raf-1 kinase, mitogen-activated protein kinase (MAPK) kinase, and MAPK, p90rsk. Inhibition of MAPK and p90rsk activation further inhibited the phosphorylation of nuclear transcription factor CREB and then influenced the binding quantity of CREB and cAMP response element, thereby inhibiting the activity of insulin promoter. CREB = cAMP response element-binding protein, HMG-COA = 3-hydroxy-3-methyl glutaryl coenzyme A.

Gene expression is a complex and orderly process and occurs owing to the interaction between numerous transeffect factors which can identify and bind the DNA and cis-acting elements. Genomic DNA of eukaryotic organisms is present in the form of chromatin. Therefore, the study of the interaction between proteins and DNA in chromatin environment is the basic way to elucidate the mechanism of eukaryotic gene expression. CHIP is an important tool for studying the interaction of DNA–protein in vivo. It can sensitively detect the binding of target protein and specific DNA fragment.^[18]^ The only specific response element for the insulin gene currently identified is CRE.^[19]^ In this experiment, the changes of the binding activity of nuclear transcription factors (CREB) and insulin promoter (CRE) in INS-1 cells were observed by using the CHIP. The results showed that the p-CREB/DNA binding of INS-1 cells was increased after high-glucose (25 mM) stimulation, and the combination of CREB and CRE was inhibited by 50 μM atorvastatin. The Ras GTPase inhibitor was used to study whether the binding activity of CREB with CRE was inhibited or not. This was consistent with the change of p-CREB expression in the nucleus of INS-1 cells that were treated with manumycin A or atorvastatin. HMG-CoA reductase inhibitors can inhibit the activity of insulin promoter and the expression of mRNA by inhibiting the Ras pathway in pancreatic beta cell. In conclusion, without the FPP modification, Ras proteins cannot locate to the cell membrane. These inhibitors downregulated the Ras proteins by inhibiting the generation of FPP and reduced the phosphorylation of a series of downstream substrates, such as Raf-1 kinase, MEK, and MAPK, p90rsk. The inhibition of MAPK and p90rsk further inhibited the phosphorylation of nuclear transcription factor CREB and then influenced the binding quantity of CREB and CRE, thereby inhibiting the activity of insulin promoter (Fig. 9).

In this study, the inhibitory effect of atorvastatin on the Ras complex (Ras/Raf/ERK/CREB) was observed. However, statins are different in their chemical structure and lipid solubility, and whether a similar effect can be applied to other statins needs to be further studied. In addition, it was found that the inhibition effect of statins on pancreatic islet function in vitro was related to the lipid solubility.^[20]^ The clinical meta-analysis suggested that statins increased the risk of diabetes, which was not related to the degree of lipid solubility.^[21]^ Therefore, the mechanism of statins on glucose metabolism still needs to be further studied in the future.
